# Illumination/Darkness-Induced Changes in Leaf Surface Potential Linked With Kinetics of Ion Fluxes

**DOI:** 10.3389/fpls.2019.01407

**Published:** 2019-11-07

**Authors:** Jinhai Li, Yang Yue, Ziyang Wang, Qiao Zhou, Lifeng Fan, Zhiqiang Chai, Chao Song, Hongtu Dong, Shixian Yan, Xinyu Gao, Qiang Xu, Jiepeng Yao, Zhongyi Wang, Xiaodong Wang, Peichen Hou, Lan Huang

**Affiliations:** ^1^College of Information and Electrical Engineering, China Agricultural University, Beijing, China; ^2^Key Laboratory of Modern Precision Agriculture System Integration Research, Ministry of Education, Beijing, China; ^3^Key Laboratory of Agricultural Information Acquisition Technology (Beijing), Ministry of Agriculture, Beijing, China; ^4^Beijing Research Center of Intelligent Equipment for Agriculture, Beijing Academy of Agricultural and Forestry Sciences, Beijing, China

**Keywords:** electrical signal, light-induced bioelectrogenesis, periodic illumination/darkness, salt stimulation, ionic mechanisms, gray relational analysis

## Abstract

A highly reproducible plant electrical signal–light-induced bioelectrogenesis (LIB) was obtained by means of periodic illumination/darkness stimulation of broad bean (*Vicia faba* L.) leaves. By stimulating the same position of the same leaf with different concentrations of NaCl, we observed that the amplitude and waveform of the LIB was correlated with the intensity of stimulation. This method allowed us to link dynamic ion fluxes induced by periodic illumination/darkness to salt stress. The self-referencing ion electrode technique was used to explore the ionic mechanisms of the LIB. Fluxes of H^+^, Ca^2+^, K^+^, and Cl^−^ showed periodic changes under periodic illumination/darkness before and after 50 mM NaCl stimulation. Gray relational analysis was used to analyze correlations between each of these ions and LIB. The results showed that different ions are involved in surface potential changes at different stages under periodic illumination/darkness. The gray relational grade reflected the contribution of each ion to the change in surface potential at a certain time period. The ion fluxes data obtained under periodic illumination/darkness stimulation will contribute to the future development of a dynamic model for interpretation of electrophysiological events in plant cells.

## Introduction

Electrical signals in plants are responsive to environmental stimuli and transmit information from the cell level to the entire plant body (Fromm and Lautner, 2007; Gilroy et al., 2018; Vodeneev et al., 2018). Although their role is similar to electrical activity in animals, plants lack a nervous system. Consequently, plants and animals show fundamental differences in signal generation, propagation, and ionic mechanisms. Fortunately, these differences do not limit the ability of plants to sense stress with induced electrical signals (Christmann and Grill, 2013; Zebelo and Maffei, 2016). Plant electrical signals are mainly distinguished as either an action potential (Trebacz et al., 2006; Galle´ et al., 2015; Zhao et al., 2015), variation potential (Stahlberg et al., 2006; Zhao et al., 2014; Vodeneev et al., 2015), local electrical potential (Opritov et al., 2005; Wang et al., 2009), or system potential (Zimmermann et al., 2009). Compared with the electrical signals in animals, spontaneous electrical signals are known in plants but are rare (Stolarz and Dziubinska, 2017). Therefore, a superior means to record plant electrical activity is by triggering signals with external stimuli, e.g., burning (Dziubinska et al., 2003), wounding (Nguyen et al., 2018), electrical (Mousavi et al., 2014), and cold stimuli (Fromm and Bauer, 1994). However, owing to differences in stimulation intensity, duration, and portion, it is challenging to achieve precise reproducibility of waveform morphology for plant electrical signals, which thus renders interpretation of electrophysiological data difficult. Therefore, the development of a simple method to obtain stable and reproducible plant electrical signals, and interpretation of the electrophysiological information carried in the waveforms have long attracted the interest of electrophysiologists.

Light is among the most important environmental factors that affect plant growth. On exposure to illumination and darkness, the leaves exhibit periodic opening and closing movements. This phenomenon is caused by changes in ion permeability of the plasma membrane when the leaf cells are excited by light and dark stimulation. The transmembrane movement of the ions cause the cells to undergo a reversible change in turgor pressure. On the basis of this physiological response of plants, we proposed a method for obtaining reproducible leaf surface potentials using periodic illumination and darkness. This local electrical potential change that cannot propagate over long distances is termed light-induced bioelectrogenesis (LIB) (Shabala and Newman, 1999). Changes in cell membrane potential are generally regarded as the plant electrical activity at the cellular level, which are associated with the underlying changes in ion channels and proton pumps activity, whereas LIB is the result of temporal and spatial superposition of various cell membrane potential changes. In fact, changes in periodic electrical potential and activities of ion channels and proton pumps reflect the information exchanged between the plant and the external environment. Previously, many scholars have used high-intensity light as a non-invasive stimulating factor to study plant electrical activity. Trebacz and Zawadzki (1985) observed that the electrical signal generated by the plasma membrane of the liverwort *Conocephalum conicum* stimulated with high-intensity light showed the same characteristics as the action potential induced by electrical stimulation. In experiments with *Asplenium trichomanes*, light-induced plasma membrane depolarization was inhibited by the anion channel blockers anthracene-9-carboxylic acid and niflumic acid, whereas the K^+^ channel blockers TEA and Ba^2+^ increased the magnitude of light-induced plasma membrane depolarization (Szarek and Trebacz, 1999). Shabala and Newman (1999) used the microelectrode ion flux estimation technique to measure the H^+^, Ca^2+^, K^+^, and Cl^−^ fluxes induced by illumination on the mesophyll and epidermal tissues of broad bean (*Vicia faba*), and observed that the ion fluxes were associated with the changes in membrane potential. Percey et al. (2014) studied the relationship between ion transport and NaCl stress of mesophyll cells in broad bean under periodic illumination, and observed that salt stress significantly affected the ability of mesophyll cells to respond to light. A comparison of the light responses of several types of plant plasma membranes revealed that K^+^ channels play a dominant role in the response of *Eremosphaera* and *Chara*, whereas other plants relied on Ca^2+^-dependent anion channels activated on the plasma membrane (Marten et al., 2010). Szechynska-Hebda et al. (2017) emphasized the interrelation between ionic mechanisms of electrical activity and regulation of photosynthesis, and proposed a light-dependent electrical signal propagation mechanism model, in which encoded ion channels and proteins are involved in the regulation of photo-responsive gene activity.

The aforementioned works, which combine light and plant electrical activity, provide inspiration for our research, and also provide a foundation for modeling plant electrical signals based on ionic mechanisms. By establishing a mathematical model of plant electrical activity, researchers can effectively analyze data and explain physiological phenomena. A number of models to simulate the electrical activity in plants have been proposed. Most early modeling studies in plants used a Hodgkin–Huxley equation to explore the changes in plant membrane potential and the mechanism of electrical signal generation (Vanduijn, 1993; Kolb et al., 1995). Subsequently, models for the propagation of action potential (Mummert and Gradmann, 1991; Sukhov et al., 2011) and variation potential (Sukhov et al., 2013) were developed. In addition, complex plant electrical signal models associated with ion transport and external stimuli have been proposed (Gradmann, 2001b, Beilby and Al Khazaaly, 2016, Novikova et al., 2017). Previous modeling work mainly used patch clamping, intracellular recording, and physiological and biochemical data to quantitatively describe the electrical signal mechanism and signal propagation.

The light-induced periodic potential, as a local potential change, can also reflect the physiological status of the plant. To investigate the mechanism of local response to external stimuli, changes in surface potential and ion fluxes induced by periodic illumination/darkness under salt stimulation were measured *in situ* in leaves of broad bean seedlings and analyzed quantitatively. In this study, we used extracellular recording to obtain the surface potential data and acquired the corresponding ion fluxes data by means of the self-referencing ion electrode technique (SIET). The present data will support future modeling studies of plant electrical signals.

## Materials and Methods

### Plant Materials and Growth Conditions

Broad bean (*Vicia faba* L.) seeds were sterilized in 10% sodium hypochlorite solution for 5 min, then rinsed in deionized water. The seeds were then soaked in deionized water (six seeds per beaker) for 12 h. The soaked seeds were placed on germination paper in a Petri dish (diameter 15 cm). The germination paper and deionized water were replaced daily. The seeds germinated after approximately 3 days. The germinated seeds were planted in 0.5 L plastic pots that contained a mixture of turfy soil, perlite, and vermiculite (3:1:1, v/v/v). The broad bean seedlings were grown in an air-conditioned room (6°C, 14 h/10 h light/dark and 60% relative humidity) and were watered four times a week. After 3 weeks, the seedlings were used in the experiments.

### Fabrication of Surface Potential Recording Electrodes

A 4-cm length of silver wire was welded onto a 20-cm-long soft thin wire. Next, stable silver/silver-chloride (Ag/AgCl) electrodes were prepared. The Ag/AgCl wire passed through a plastic annulus with a hole (diameter 1 mm) on its lower middle part and the hole was sealed using hot-melt adhesive. The annulus was open at both ends, with diameter 25 mm, height 20 mm, and wall thickness 1 mm. Agarose solution (50 ml; 0.5% agar and 10 mM KCl) was heated using a magnetic stirrer until the agar was completely dissolved. Boiling agarose solution (2 ml) was injected into the plastic annulus, which was placed flat on a Petri dish. It should be noted that the Ag/AgCl wire must be completely embedded in the agarose solution. After solidification of the agarose solution in the plastic annulus, the device could be used as a recording electrode of surface potential.

### Acquisition of LIB

**Figure 1:**
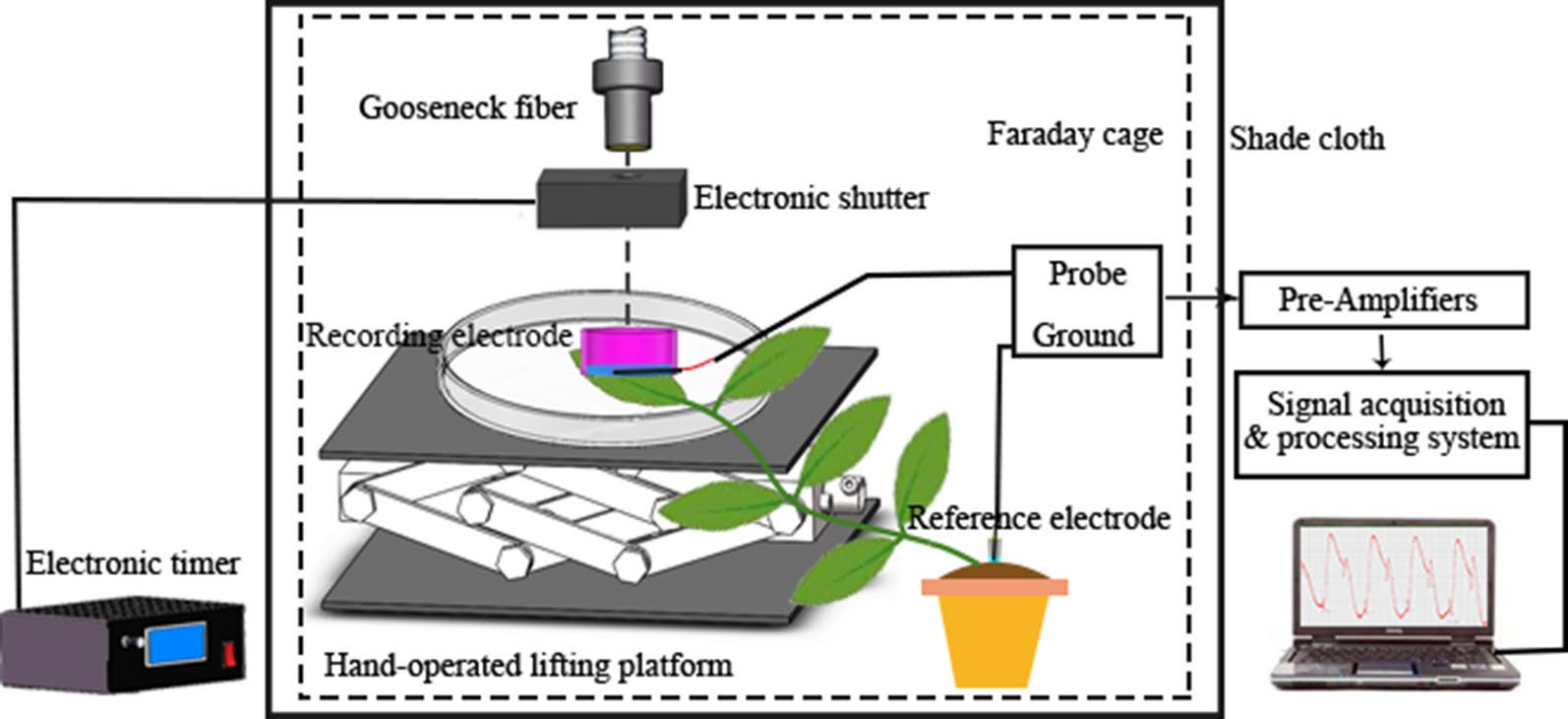
Schematic diagram of the system for electrical signal acquisition.

The youngest mature leaf on a broad bean seedling was placed flat on the bottom of a Petri dish (diameter 10 cm), and the agar gel of a recording electrode was placed in close contact with the leaf surface. The reference electrode was constructed by immersing an Ag/AgCl electrode into a pipette containing 0.2% agar and 5 mM KCl, and then inserted into the soil of the plastic pot containing the seedling (Mousavi et al., 2014). Two hours before recording, the seedling was watered to ensure that the reference potential remained stable when recording electrical signals. The recording electrode and the reference electrode were connected to the input terminal and the ground terminal of the probe (RM6240BD, Chengdu Instrument Factory, Chengdu, China), respectively. Given that the plant electrical signal is weak, the detected original signal must pass through a high-input-impedance pre-amplifier (SWF-1B, Chengdu Instrument Factory) to be measured effectively. A tungsten halogen lamp (MHAA-100W; MORITEX, Tokyo, Japan) was used as the stimulus light source and placed outside the Faraday cage. A gooseneck optical fiber (A08400; MORITEX) passed through the cage and the end of the fiber was positioned 10 cm above the surface potential recording electrode. An electronic timer (GCL-73M; Daheng Optics, Beijing, China) that controlled an electronic shutter (GCL-7103M; Daheng Optics) was placed at the optical fiber outlet. The duration of shutter opening and closing was 600 s. The Faraday cage was covered with black shade cloth to prevent the influence of external natural light. In the experiment, the temperature was maintained at 26°C and the relative humidity was ~60%. A schematic diagram of the electrical signal acquisition system is shown in **Figure 1**.

### Salt Stimulation of LIB

In salt stimulation experiments, the conductive agar gel embedding a recording electrode was supplemented with a given concentration of NaCl (0, 50, or 250 mM). Thus, the sensor recorded salt-induced potential changes. This approach of utilizing replacement recording electrodes to study the effects of different concentrations of NaCl on the surface potential of the same broad bean leaf in an experiment did not require prolonged salt-stress treatment. The leaf responded rapidly to salt stimulation, and within 60 min the value of baseline voltage would return to a steady state. For comparability of the surface potential before and after salt stimulation, we ensured that the recording area before and after salt stimulation was consistent when changing the surface potential recording electrodes. In this work, we chose to stimulate the leaves rather than the roots, because stimulation of leaves generates a response in a short time and the effect is obvious, whereas stimulation of roots requires a higher NaCl concentration and longer processing time. From a biological point of view, this reflects that plants cannot control Na^+^ loading of the xylem, and as a result the salt tolerance of leaf tissues is affected (Wu et al., 2013).

### Ion Fluxes Measurement

The ion fluxes of H^+^, K^+^, Ca^2+^, and Cl^−^ under periodic illumination were measured using the SIET technique. The SIET, which has been applied widely in physiology, can monitor the local extracellular ion gradient and the transmembrane dynamic transport of ions in real time with high sensitivity to square-micron spatial resolution in a non-invasive manner (Smith et al., 1999; Antoine et al., 2001; Sun et al., 2009; Wu et al., 2018). The microelectrodes were made of borosilicate glass tubes without filaments (B15024F; VitalSense Scientific Instruments Co., Ltd, Wuhan, China) and subjected to silanization prior to use (Smith et al., 1999). In the process of usage, the silanized microelectrodes were back-filled with a short column of electrolyte (about two-thirds the length of the electrode), and then filled with liquid ionic exchanger (LIX). Fabrication details for the ion-selective microelectrodes used in the experiments are shown in **Table 1** (Shabala and Newman, 1999; Shabala and Shabala, 2002a; Shabala et al., 2002b; Percey et al., 2014; Xue et al., 2016).

**Table 1:** Fabrication details for the ion-selective microelectrodes containing liquid ionic exchanger (LIX) used in the study.

Ion	Electrolyte solution components	LIX	Calibration set
H^+^	15 mM NaCl + 40 mM KH_2_PO_4_, PH 6.5	Hydrogen ionophore I—cocktail A (95291; Sigma-Aldrich)	5.5–6.5–7.5 (pH)
K^+^	200 mM KCl	Potassium ionophore I—cocktail B (99373; Sigma-Aldrich)	1–2–5 (mM KCl)
Ca^2+^	100 mM CaCl_2_	Calcium ionophore I—cocktail A (99310; Sigma-Aldrich)	0.5–1–2 (mM CaCl_2_)
Cl^-^	200 mM KCl	Chloride ionophore I—cocktail A (99408; Sigma-Aldrich)	1–3–5 (mM KCl)

### Ion Flux Measurement Protocols

We used the youngest mature leaf of a 3-week-old broad bean seedling for ion flux measurement.The leaf was trimmed with scissors in a direction perpendicular to the veins. The trimmed area wasabout one-eighth of the total area of the intact leaf. The remaining portion of theleaf was immersed in a Petri dish (φ 90 mm) containing 15 ml basic solution(2 mM KCl, 1 mM CaCl_2_, pH 6.5). To prevent the leaf from moving in the basic solution,two 1.5 g pebbles were placed on opposite ends of the leaf. The stem of the broad bean seedling wasthen carefully secured to the edge of the Petri dish with Blu-Tack. Approximately 1.5 h later, the ion flux experiment was performed. The ion-selective electrode vibrated at a frequency of 0.5 Hz within a distance of 30 µm along the *x*-axis, which was approximately 3–5 µm from the leaf surface. The frequency used in the experiments was set according to the response time of the electrode. The response time is defined as the time elapsed for the electrode potential to change to 90% of the total change (Wang and Bishop, 2010). Thus, response time of each ion selective microelectrode in this work according to the method in the literatures (Wang and Bishop, 2010; Xue et al., 2016). We tested eight times for each ion-sensitive microelectrode, and then the response time of each ion selective microelectrode was shown in **Supplementary Table 1**. With regard to cell types in the stimulated leaf, the microelectrode was mainly confocal with mesophyll cells, but the trimmed leaf also included a large number of epidermal cells and guard cells. For salt stimulation, the basic solution in the Petri dish was removed with a pipette, and 15 ml basic salt solution (2 mM KCl, 1 mM CaCl_2_, 50 mM NaCl, pH 6.5) was added. The ion flux recorded was the net flux of several cell types in the measurement area. In addition, we measured the corresponding membrane potential by means of extracellular recording. The recorded potential is the result of spatial superposition of the membrane potentials of the cell population.

**Figure 2:**
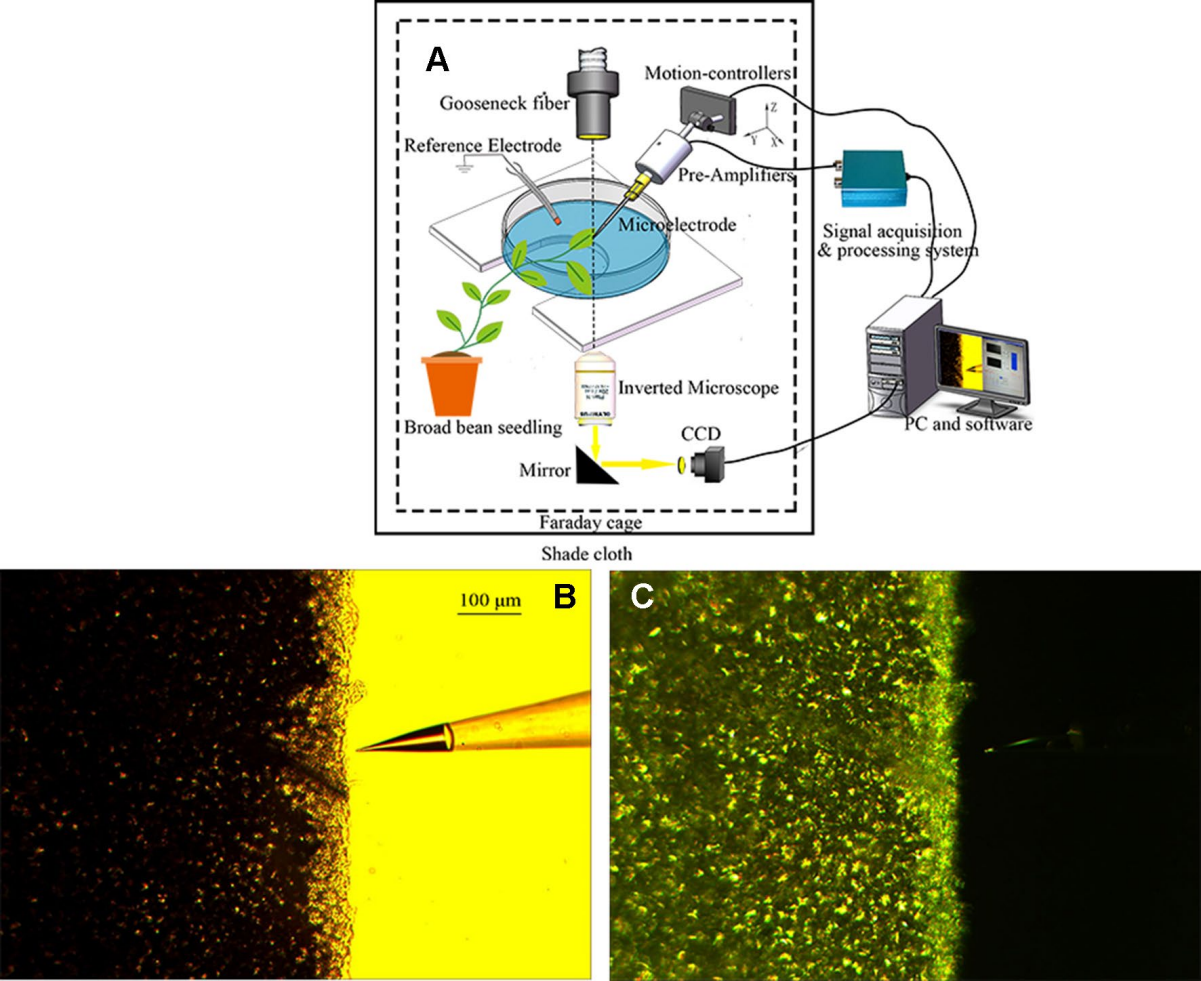
Ion flux measurement in the leaf of a broad bean seedling under periodic illumination/darkness. **(A)** Schematic diagram of ion flux measurement. **(B**, **C)** Measurement of H^+^ flux under periodic illumination/darkness.

Given that ion flux measurement is susceptible to external noise interference, the experiment was performed in a Faraday cage. Before the experiment, the gooseneck fiber through the Faraday cage was placed directly above the leaf, and the photoperiod was 20 min (10 min light/10 min dark). The light was provided by a tungsten halogen lamp through an optical fiber, which was positioned 10 cm from the leaf. The photon flux density (PFD) was 278 µmol·m^−2^·s^−1^. The Faraday cage was covered with black shade cloth to prevent the influence of ambient natural light, and the PFD after covering was 0 µmol·m^−2^·s^−1^. The system for ion flux measurement in the leaf of a broad bean seedling under periodic illumination/darkness is shown in **Figure 2**.

### Gray Relational Analysis Applied to Ion Fluxes Data Analysis

By definition, systems with fully known, partially known, and fully unknown information are referred to as white, gray, and black systems, respectively (Deng, 1982; Deng, 1989; Kuo et al., 2008; Verma and Pandey, 2018). The gray theory was proposed by Deng (Deng, 1982; Deng, 1989), which included gray relational analysis (GRA). GRA is an effective method to analyze uncertain correlations between things and system factors, or factors and the main behavior, and enable investigation of quantitative and qualitative relationships using relatively little data or with considerable variability in factors (Deng 1982; Deng, 1989; Wang et al., 2009; Liu and Lin, 2010; Zhang and Zhang, 2013; Guo and Kang, 2018). In addition, because there is no restriction on the type of samples and the probability distribution, GRA has been widely applied in diverse fields of research (Wang et al., 2009; Zhang and Zhang, 2013; Guo and Kang, 2018; Sun et al., 2018; Wang et al., 2018).

In the present work, GRA was applied to evaluate the relations between surface potential changes and ion fluxes to determine the main factors in a given period. The gray relational coefficient *ξ*[*k*], gray relational grade (GRD) R_0_*_i_*(*x*_0_, *x_i_*), and gray relational ordering are defined as follows. Before conducting GRA, data pre-processing was performed for normalization of the raw data for analysis (Nain et al., 2018).

Let *x* = {*x_i_* | *i*∈I} be a space sequence where *i* = (1,2,…*m*). *x_i_* is a factor of the system, and its value at the *k*th entity in the sequence is *x_i_* [*k*], where *k* = 1,2,…,*n*. If we denote the reference sequence *x*_0_ by *x*_0_ (1), *x*_0_ (2),…, *x*_0_ (*n*), i.e. the observed surface potential changes in a plant, and the compared sequence *x_i_* by *x_i_* (1), *x_i_* (2),…, *x_i_* (*n*), *x_i_*, i.e. ion fluxes, then the gray relational coefficient between *x*_0_ and *x_i_* at the *k*th entity is obtained by Eq. (1)

The gray relational coefficient *ξ*[*k*] indicates the relational grade between two series at a certain time point. The GRD, R_0_*_i_*(*x*_0,_*x_i_*), represents the degree whereby two series can be correlated. It describes the trend relationship between the reference series and a compared series in the system.

(1) $$\xi_{i}[k]=\frac{\underset{i}{\text{min}}\underset{k}{\text{min}}\left|x_{0}[k]-x_{i}[k]\right|+\underset{i}{\text{max}}\underset{k}{\text{max}}\left|x_{0}[k]-x_{i}[k]\right|}{\left|x_{0}[k]-x_{i}[k]\right|+\underset{i}{\text{max}}\underset{k}{\text{max}}\left|x_{0}[k]-x_{i}[k]\right|}$$

(2) $$R_{0i}(x_{0},x_{i})=\frac{1}{n}\sum_{k=1}^{n}\xi_{i}[k]$$

where *i* (= 1,2,3,…,*m*) is the factor; *k* (= 1,2,3,…*n*) is the time point; *x*_0_[*k*] is the reference series; x*_i_*[*k*] is the compared series; [insert math(3) here] denotes the absolute difference between the two sequences; and [insert math(4) here] and [insert math(5) here] are the minimum and maximum values, respectively, of the absolute differences in all compared sequences.

One of the main purposes of GRA is to find the importance order of the factors based on the values of R_0_*_i_*(*x*_0,_*x_i_*) and identify the key factors affecting electrical signals in plants. Thus, the rankings of the relational grades (gray relational ordering) are more significant than the values of GRDs between factors. If *R*_0_*_i_*>*R*_0_*_j_*, then the influence of *x_i_* on *x*_0_ is more dominant than that of *x_j_* on *x*_0_. If *R*_0_*_i_* = *R*_0_*_j_*, then the influence of *x_i_* on *x*_0_ is equal to that of *x_j_* on *x*_0_.

The gray relational coefficient *ξ*[*k*] indicates the relation grade between two series at a certain time point. The gray relation grade R_0_*_i_*(*x_0_*,*x_i_*) represents the degree whereby two series can be correlated. It describes the trend relationship between the reference series and a compared series in the system.

## Results

### Light-Induced Bioelectrogenesis

**Figure 3:**
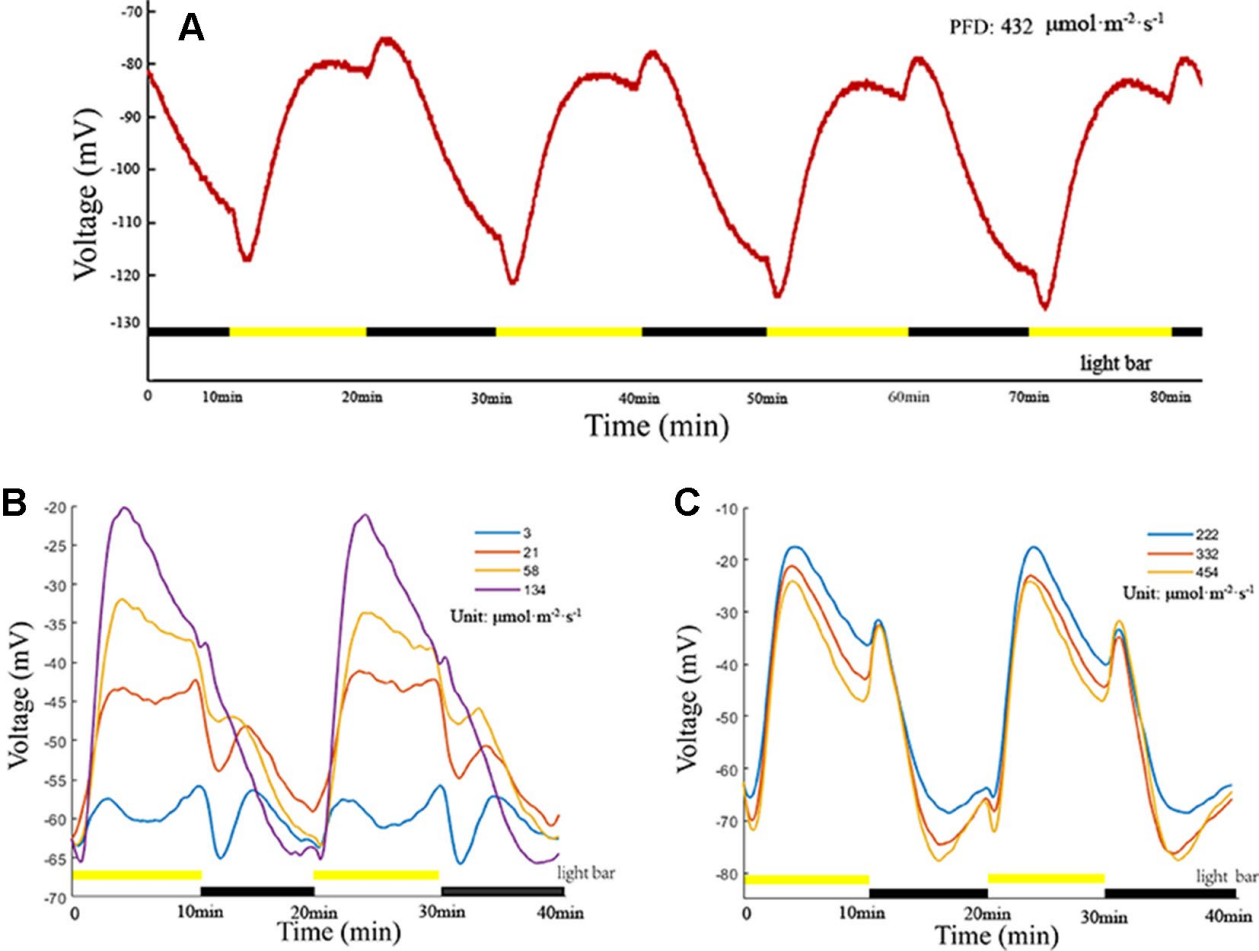
Light-induced bioelectrogenesis (LIB). **(A)** A highly reproducible LIB under periodic illumination/darkness. The yellow bars indicate the illumination periods and the black bars indicate the darkness periods. **(B**, **C)** LIB of different amplitudes under different light intensities. Yellow bars and black bars are not in the same line, indicating that the light intensity was adjusted.

LIB is a highly reproducible plant electrical signal, and its waveform, amplitude, and variation are illumination intensity-dependent and duration-dependent (**Figures 3A–C**). In the present experiment, all photoperiods were of 20 min duration (10 min light/10 min dark). When the PFD attained 3 µmol·m^−2^·s^−1^, an extremely low amplitude periodic plant electrical signal was generated (**Figure 3B**). With the increase in light intensity, the amplitude of the electrical signal increased, and the shape of the waveform gradually changed. When the PFD attained 222 µmol·m^−2^·s^−1^, the amplitude and waveform assumed a stable state (**Figure 3C**). With continued increase in the light intensity, the amplitude and waveform did not change notably. These findings suggested that the net ion fluxes attained a stable state at PFD of 222 µmol·m^−2^·s^−1^. When the light intensity attained a certain value (between 58 and 134 µmol·m^−2^·s^−1^ in the series presented), the instantaneous light stimulation caused the surface potential to decline rapidly, lasting for about 35 s, and then returned gradually to the original amplitude. Subsequently, the surface potential entered a relatively rapid rising phase. Repeated experiments on broad bean seedlings (n = 20, n—number of seedlings) showed that the time of the rising phase was relatively stable (~200 s), which did not change dramatically with the change in light intensity. With extension of the duration of illumination, the waveform entered a phase of relatively slow descent until the illumination stage ended. During the illumination stage, the surface potential was not restored to the initial potential value before illumination and after declining to a certain value it entered a phase of relative stability. After imposition of sudden darkness, the surface potential showed a small rising phase, and then entered a prolonged falling phase until the initial potential before illumination was attained. Although it is difficult to maintain a consistent baseline value of the surface potential owing to the influence of the reference potential, contact potential, and individual plant differences, the peak-to-peak values of waveforms were similar and waveforms had the same changing trend under the same illumination conditions (**Figures 3A–C**).

### Effects of Salt Stimulation on LIB

**Figure 4:**
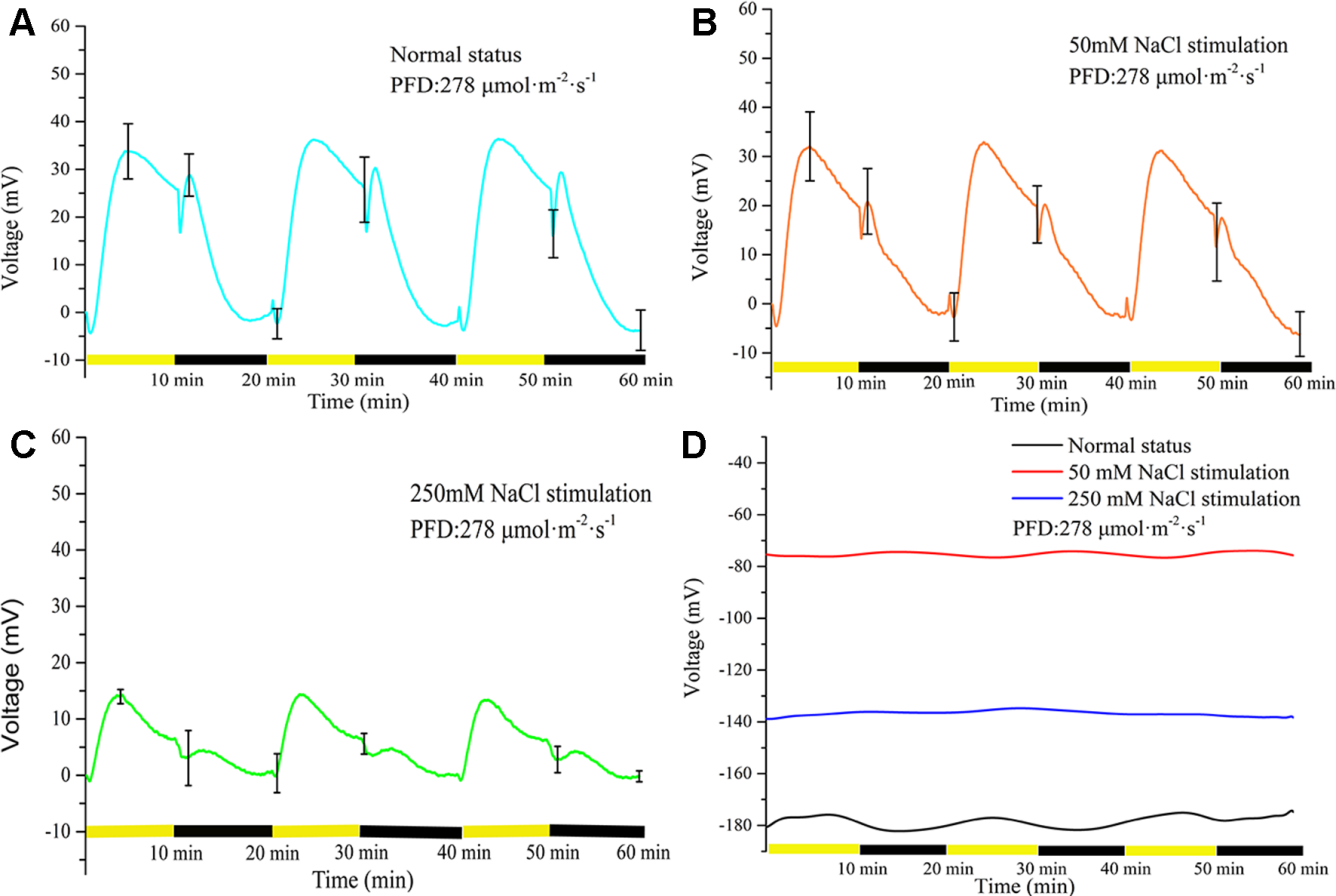
Effects of three concentrations of NaCl on light-induced rhythmical bioelectrogenesis. With increase in NaCl concentration, the amplitude of the surface potential at the same position of the same leaf gradually decreased and the waveform also varied considerably. The results indicated that the leaf response to light gradually decreased with increase in salt stimulation intensity. The potential change without leaf under illumination/darkness was shown in **Supplementary Figure 3**. The leaves used in **(A**–**C)** were living, whereas a heat-killed leaf was used in **(D)**. To make statistical analysis on the amplitude of the voltage, the baseline values in **(A**–**C)** were subtracted. Mean ± SE (*n* = 6); n—number of seedlings.

Three NaCl concentrations (0, 50, and 250 mM) were used to investigate the effect of salt stimulation on the periodic light/dark-induced surface potential. The NaCl concentration was correlated with the light-induced periodic electrical potential generated by the leaf. With increase in the NaCl concentration, the amplitude of potential generated by the same leaf significantly decreased (**Figure 4**). After leaf inactivation, LIB no longer changed drastically with the increase in NaCl concentration (**Figure 4D**). When a leaf is stimulated by salt, the ionic homeostasis of the leaf tissue is disturbed, which changes the ionic permeability of the plasma membrane (Shabala and Newman, 2000). After 60 min salt stimulation, the transmembrane movement of ions restored a new stable state, and then the surface potential showed a highly reproducible periodic change again. The time required for ions to restore the new stable state varied with the intensity of the stimulus; the higher the stimulus intensity, the longer taken by ions to attain homeostasis, but generally it was achieved within 60 min.

### Kinetics of Net H^+^, Ca^2+^, K^+^, and Cl^–^ Fluxes Under Periodic Illumination/Darkness

In the present work, periodic illumination/darkness changes induced leaves to generate a slowly changing electrical signal. However, the fluctuations did not propagate from the stimulated leaf to other leaves. The fluctuation was considered to be a change in local potential, i.e. a macroscopic representation, which involved activity of ion channels and proton pumps at the microscopic level. Although researchers have long been interested in changes in plasma membrane ion transport caused by light stimulation, the ionic mechanisms generated by light-dependent signals with salt stimulation is worthy of careful study. We used the SIET to record the ion fluxes under periodic illumination/darkness, and to examine the association of the kinetics of H^+^, Ca^2+^, K^+^, and Cl^–^ fluxes with periodic surface potential. Under the NaCl stimulation condition, the net fluxes of all four ions were changed, but the majority still showed periodic changes. In addition, in order to improve the accuracy of ion fluxes data, we verified whether there was a voltage difference between the two positions of electrode in the basic solution without leaf sample under illumination/darkness (**Supplementary Figure 1**).

#### Measurement of H^+^ Flux Under Periodic Illumination/Darkness

**Figure 5:**
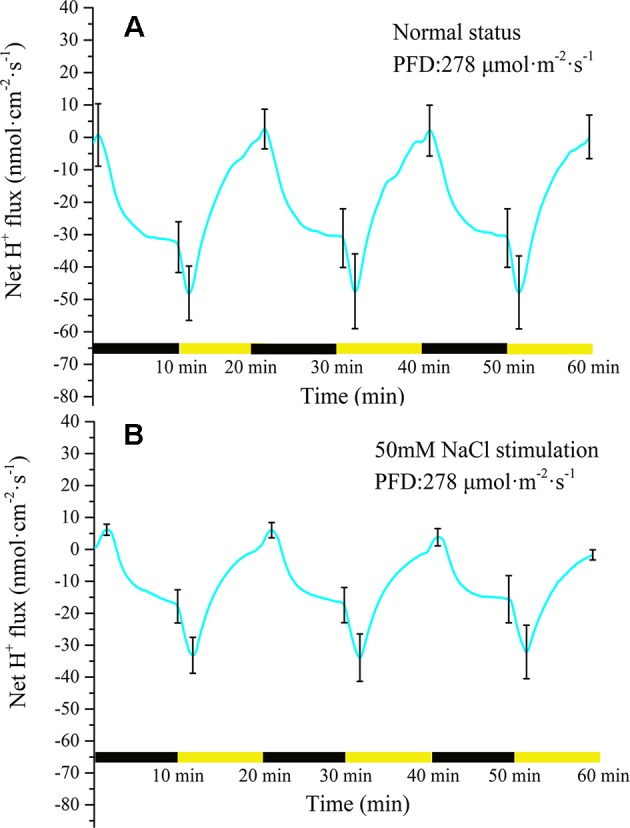
Measurement of net H^+^ flux before and after 50 mM NaCl stimulation. **(A)** Net H^+^ flux induced by periodic illumination/darkness without NaCl treatment (normal condition). The H^+^ flux exhibited distinct periodic changes induced by periodic illumination/darkness stimulation. **(B)** Net H^+^ flux induced by periodic illumination/darkness with 50 mM NaCl stimulation. The efflux of H^+^ was significantly reduced and the influx significantly increased after 50 mM NaCl stimulation. Note: wavelet decomposition was applied to the raw H^+^ flux data to remove noise using the Daubechies (db4) wavelet transform, where the wavelet layer number is 3–4. Mean ± SE (*n* = 5); n—number of seedlings.

Measurement of H^+^ flux provided evidence that the proton pump may be involved in the electrical activities of the plant. Under the periodic illumination/darkness stimulation, the H^+^ flux exhibited distinct periodic changes. The ion fluxes data is inevitably interfered by external noise when it is acquired, and then the signal contains a lot of noise accordingly. To extract the trend of the signal, the raw data must be denoised. Wavelet transform is a common signal processing method (Unser and Aldroubi, 1996; Mallat, 1999). It performs multi-scale refinement analysis of functions or signals through computational functions such as scaling and translation, effectively extracting accurate information from the original signal, e.g., patch clamp signals (Bruce and Larsen, 2000; Diserbo et al., 2000; Chen et al., 2007; Kargol, 2013). As shown in **Supplementary Figure 2**, wavelet transform was used to eliminate the effect of noise. For the majority of the measurement period H^+^ was in an efflux state (**Figure 5A**). With the switch from darkness to illumination, the H^+^ efflux rapidly increased from 32 nmol cm^−2^ s^−1^ to 48 nmol cm^−2^ s^−1^ over a period of approximately 90 s. With extension of illumination, the efflux of H^+^ continued to decrease and attained an influx state before the switch to darkness. At the beginning of the darkness stage, the influx of H^+^ continued to increase. After approximately 30 s, the influx of H ^+^ peaked (~2.5 nmol cm^−2^ s^−1^) and thereafter decreased. After a few minutes, H^+^ efflux was observed again. With extension of darkness, the efflux of H^+^ increased rapidly and then gradually stabilized (~35 nmol cm^−2^ s^−1^).

Compared with the normal condition (without NaCl stress as the control), 50 mM NaCl treatment significantly decreased the efflux of net H^+^ under periodic illumination/darkness (**Figure 5B**). The influx of H^+^ increased slightly and the efflux decreased significantly under 50 Mm NaCl stimulation. Normally, the ion fluxes after salt stimulation will attain a steady state again after 20 min. In the present experiment, all ion fluxes data after NaCl stimulation were collected after 30 min. Similar to before salt stimulation, the efflux of H^+^ increased dramatically after the switch from darkness to illumination (from 17 to 33 nmol cm^−2^ s^−1^). After illumination for approximately 90 s, the efflux of H^+^ peaked. With extension of illumination, the H^+^ efflux continued to decrease and attained influx before the switch to darkness. Compared with the normal condition, the duration of H^+^ influx increased significantly to 120 s.

#### Measurement of Ca^2+^ Flux Under Periodic Illumination/Darkness

**Figure 6:**
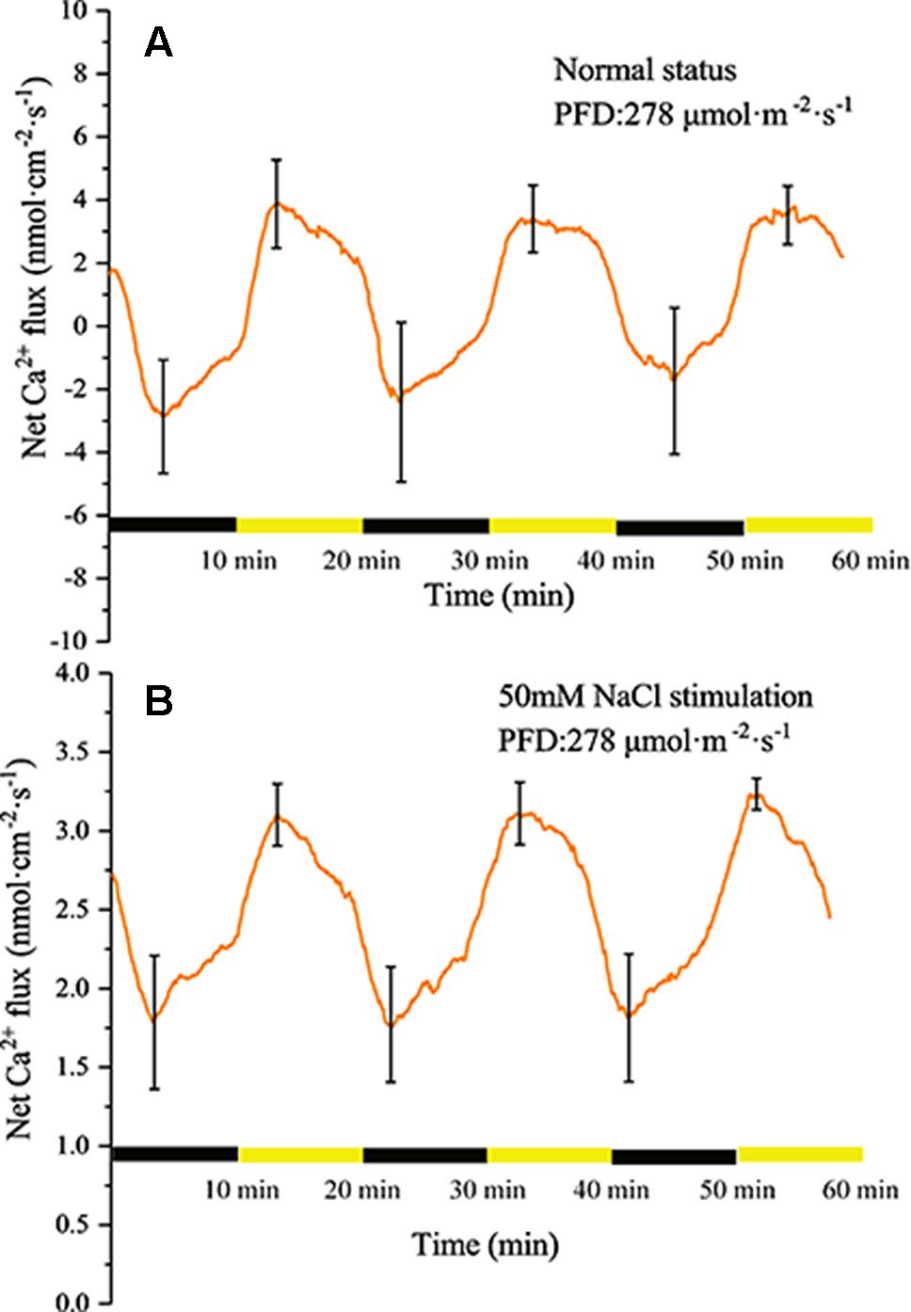
Measurement of net Ca^2+^ flux before and after 50 mM NaCl stimulation. **(A)** Net Ca^2+^ flux induced by periodic illumination/darkness without NaCl treatment (normal condition). **(B)** Net Ca^2+^ flux induced by periodic illumination/darkness with 50 mM NaCl stimulation. Note: wavelet decomposition was applied to the raw Ca^2+^ flux data to remove noise using the Daubechies (db4) wavelet transform, where the wavelet layer number was 2–4. Mean ± SE (*n* = 4); n—number of seedlings.

Similar to the H^+^ flux, Ca^2+^ also exhibited changes under periodic illumination/darkness (**Figures 6A, B**). At the start of the illumination stage, the net Ca^2+^ flux gradually changed from efflux to influx and peaked (~3.9 nmol cm^−2^ s^−1^) over approximately 4 min, then decreased at a faster rate. At the start of the dark phase, the influx of Ca^2+^ continued to decrease and was converted into efflux over approximately 2 min, and peaked (~2.8 nmol cm^−2^ s^−1^) over approximately 4 min.

Compared with the normal condition, the transmembrane transport state of Ca^2+^ changed notably after stimulation with 50 mM NaCl. The influx of Ca^2+^ increased significantly, whereas the efflux was abolished (**Figure 6B**).

#### Measurement of K^+^ Flux Under Periodic Illumination/Darkness

**Figure 7:**
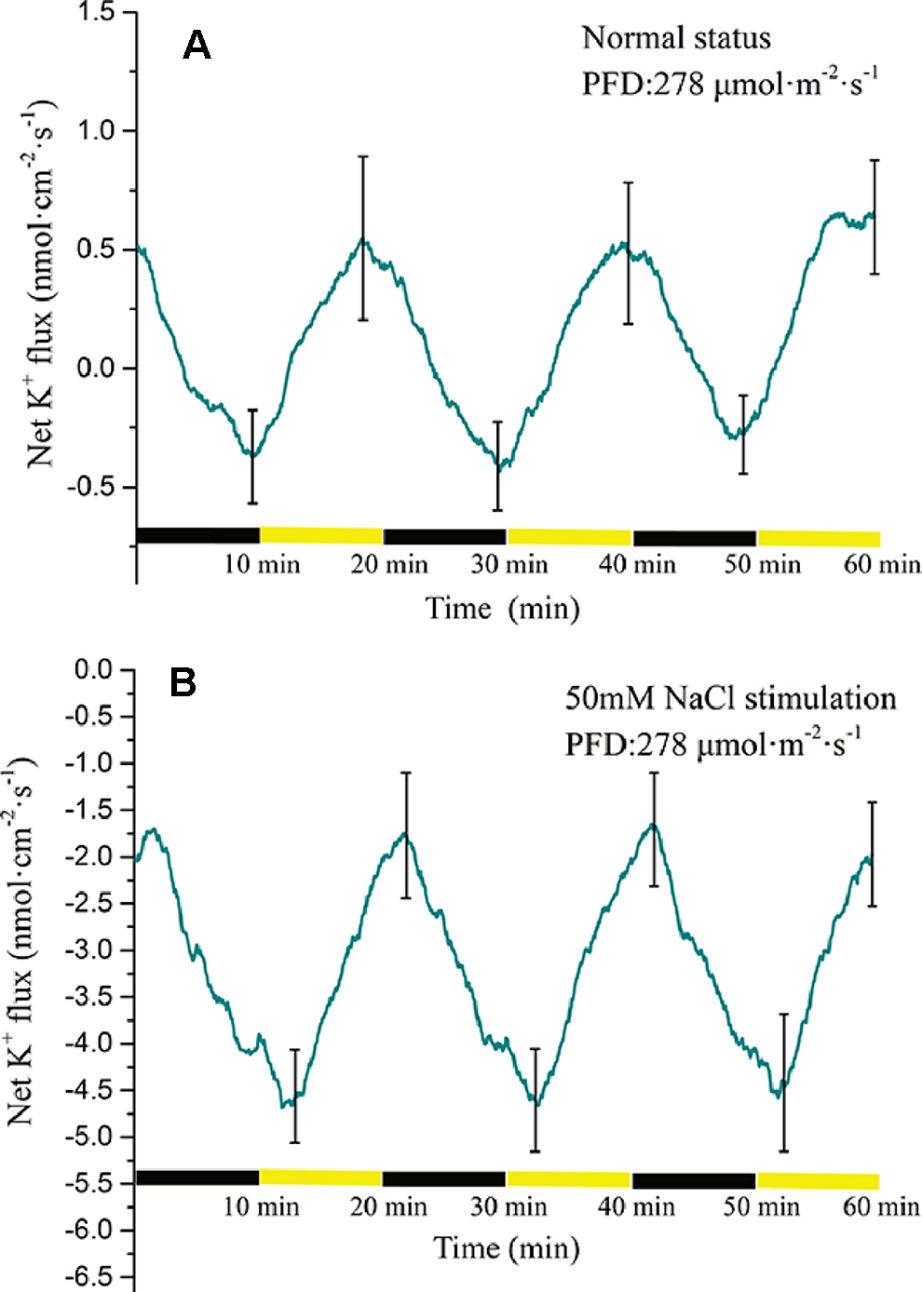
Measurement of net K^+^ flux before and after 50 mM NaCl stimulation. **(A)** Net K^+^ flux induced by periodic illumination/darkness without NaCl treatment (normal condition). **(B)** Net K^+^ flux induced by periodic illumination/darkness with 50 mM NaCl stimulation. The duration of net K^+^ influx significantly increased after 50 mM NaCl stimulation. Negative values represent ion efflux. Note: wavelet decomposition was applied to the raw K^+^ flux data to remove noise using the Daubechies (db4) wavelet transform, where wavelet layer number was 2–4. Mean ± SE (*n* = 4); n—number of seedlings.

The K^+^ flux displayed a periodic change in response to periodic illumination/darkness (**Figure 7A**). Before NaCl stimulation, the net K^+^ flux showed alternating states of influx and efflux. Under darkness, the influx of K^+^ decreased gradually and was converted into efflux over approximately 3 min. The duration of K^+^ efflux was about 8 min. Under illumination, the K^+^ efflux initially increased and thereafter decreased gradually. The influx of K^+^ was initiated about 3 min after the start of illumination. With 50-mM NaCl stimulation, the K^+^ flux differed notably from that in the normal condition (**Figure 7B**). The net K^+^ flux remained in an efflux state. In the darkness stage, the efflux of K^+^ initially decreased and peaked (~1.7 nmol cm^−2^ s^−1^). Thereafter, the efflux gradually increased with extension of darkness. Under illumination, the K^+^ efflux continued to increase and peaked over approximately 3 min (~4.5 nmol cm^−2^ s^−1^). With extension of illumination, the efflux of K^+^ decreased gradually.

#### Measurement of Cl^−^ Flux Under Periodic Illumination/Darkness

**Figure 8:**
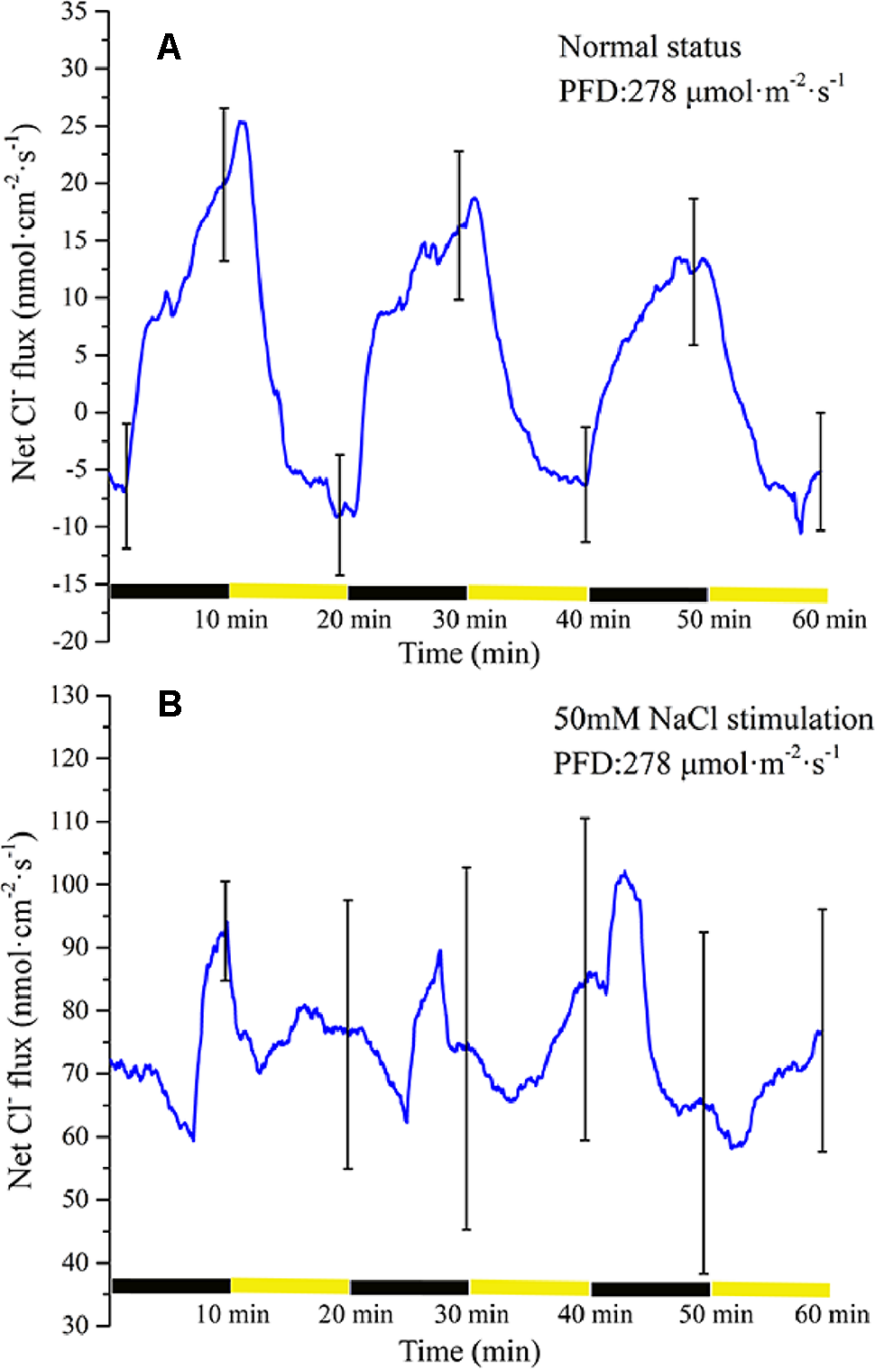
Measurement of net Cl^−^ flux before and after 50 mM NaCl stimulation. **(A)** Net Cl^−^ flux induced by periodic illumination/darkness without NaCl stimulation (normal condition). **(B)** Net Cl^−^ flux induced by periodic illumination/darkness with 50 mM NaCl. Negative values represent ion efflux. Note: wavelet decomposition was applied to the raw Cl^−^ flux data to remove noise using the Daubechies (db4) wavelet transform, where wavelet layer number was 1–4. Mean ± SE (*n* = 3); n—number of seedlings.

Periodic illumination induced a periodic change in net Cl^−^ flux (**Figure 8A**). Under illumination, the influx of Cl^−^ gradually attained the maximum value. Thereafter, the influx of Cl^−^ decreased gradually. About 3 min later, the net Cl^−^ flux began to efflux. Under darkness, the efflux of Cl^−^ continued to increase and peaked. About 7 min later, the net Cl^−^ flux was converted into an influx.

The net Cl^−^ flux in response to periodic illumination/darkness with 50 mM NaCl stimulation (**Figure 8B**) differed from that in the normal condition (**Figure 8A**). After NaCl stimulation, a significant change in Cl^−^ flux was observed. When stimulated by 50-mM NaCl, substantial Cl^−^ flux was recorded, whether under illumination or darkness, without exhibiting efflux, and the peak influx was several times that before salt stimulation.

#### Gray Relational Grade between Each Ion Flux and LIB

Under periodic illumination/darkness, periodic changes in LIB involved dynamic changes in ion transmembrane transport. The calculated GRDs in a certain time period are presented in **Table 2**. Accordingly, the GRD values were ranked based on the gray relational degree from large to small in different periods.

**Table 2:** Gray relational grades between each ion flux and surface potential under illumination and darkness.

Light on		Light off	
Rising phase	Falling phase	Rising phase	Falling phase
Ca^2+^ 0.6625	Ca^2+^ 0.8681	Ca^2+^ 0.5622	Ca^2+^ 0.5514
Cl^−^ 0.8511	Cl^−^ 0.7640	Cl^−^ 0.4773	Cl^−^ 0.4663
K^+^ 0.6070	K^+^ 0.7939	K^+^ 0.5002	K^+^ 0.7166
H^+^ 0.5347	H^+^ 0.7208	H^+^0.5230	H^+^ 0.8377
Cl^-^ > Ca^2+^ > K^+^ > H^+^	Ca^2+^ > K^+^ > Cl^-^ > H^+^	Ca^2+^ > H^+^ > K^+^ > Cl^-^	H^+^ > K^+^ > Ca^2+^ > Cl^-^

## Discussion

### Electrophysiological Significance of LIB

Fluctuations in surface potential are the result of the space–time superposition of membrane potential changes in the population cells (mesophyll cells, epidermal cells, and guard cells) that are in contact with the recording electrode (Huang et al., 2010; Zhao et al., 2015). Thus, the LIB differs from the membrane potential of a single type of mesophyll cell induced by light. The amplitude of LIB is lower than that of the membrane potentials induced by light stimulation of individual mesophyll cells, which may be due to each individual cell showing a different polarization state or polarization amplitude under the same illumination conditions. The LIB is a local electrical signal that cannot be transmitted over long distances (which has previously been proven experimentally and is not discussed here). Plasma membranes of mesophyll cells and epidermal cells depolarize under strong light stimulation, whereas the plasma membrane of guard cells exhibit hyperpolarization. However, the depolarization amplitude of the plasma membrane of mesophyll cells is much larger than that of the plasma membrane of epidermal cells (Shabala and Newman, 1999; Živanović et al., 2005). Fortunately, the waveform of LIB is also associated with factors such as light intensity, photoperiod, and light quality. As for the effect of light intensity on LIB, we have verified it through experiments (**Figures 3B, C**) and the effect of photoperiod on LIB has been mentioned in the article published by our research group (Wang et al., 2019). The effect of light quality on LIB will be another meaningful work. Light quality (spectrum/wavelengths of perceived irradiance) influences regulation of plasma membrane, which are associated with the presence of plant photoreceptors such as blue and red-light receptors. Usually different types of cells respond differently to light quality. For instance, there is evidence that blue light can activate H^+^-ATPase on the plasma membrane of the guard cell, causing the drastically efflux of H^+^ to activate the inward rectifier potassium channel, which in turn causes hyperpolarization of the plasma membrane (Marten et al., 2010; Uozumi and Schroeder, 2010; Kollist et al., 2014). But on illumination with blue light, voltage-dependent and calcium-permeable channels activate in the plasma membrane of mesophyll cells, causing depolarization of the plasma membrane (Dharmawardhane et al., 1989; Stoelzle et al., 2003). In some researches, red light is also thought to cause bioelectrical events (Lee and Bowling, 1995; Mott et al., 2008; Kisnierienė et al., 2014). LIB is a superimposed potential of different types of cells, and light quality change will inevitably affect its waveform. The effect of light quality and photoperiod on surface potential is a topic worthy of further in-depth research and controlling these variables enables the recording of surface potentials of improved stability.

The LIB carries abundant electrophysiological information, and the waveform and amplitude of the surface potential changes significantly when the leaf is stimulated by salt. The waveform and amplitude of the surface potential recorded at the same location of the same leaf were changed under different concentrations of NaCl. This is mainly because different concentrations of salt have different effects on ion transmembrane transport, and thus plants respond differently to light. It is fascinating that this change can be demonstrated in the early stages of salt stress without the need for long-term assessment of morphological phenotypes or measurement using complex chemical methods. In combination with our previous work (Wang et al., 2019), this highly reproducible electrical signal may be useful to identify whether a plant is suffering salt stress.

### Ionic Mechanisms of LIB

**Figure 9:**
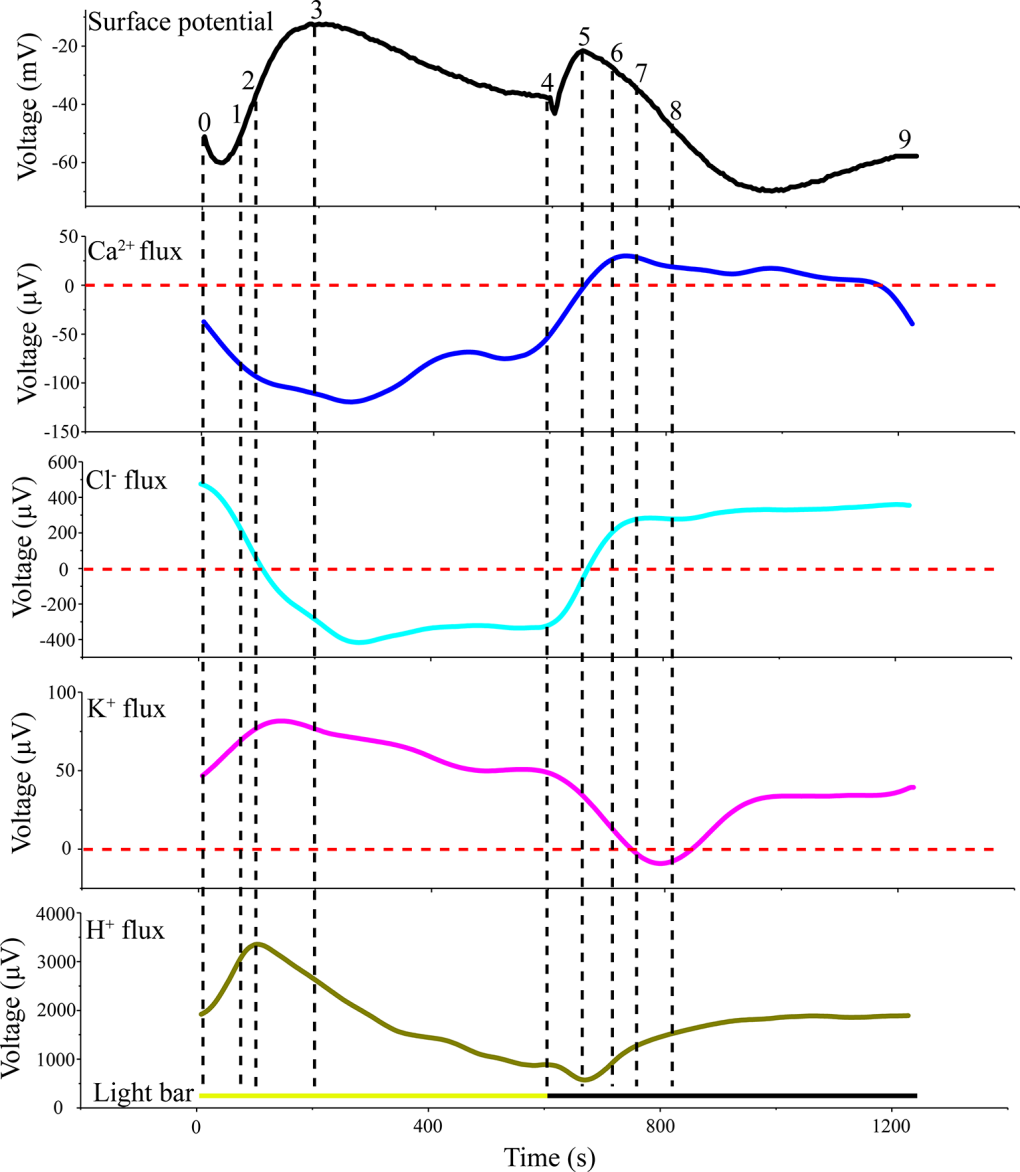
Original voltage difference values of H^+^, Ca^2+^, K^+^, and Cl^−^ and light-induced bioelectrogenesis in the same period. All ion fluxes were derived from the original value measured by SIET. The voltage difference values of the ion fluxes and surface potential were divided into nine segments (0–1; 1–2;…7–8; 8–9) by selecting crossing points (turning points) in each curve.

LIB showed slow changes in leaf surface potential triggered by periodic illumination/darkness. The transmembrane transport of ions excited by periodic illumination were reflected by LIB (**Figure 9**). Therefore, based on the acquired ion fluxes data, we discuss the ionic mechanisms of LIB (**Supplementary Table 2**).

Different types of cells in the leaf show different ionic mechanisms in response to light. Previous studies explored these mechanisms by measuring ion fluxes of different types of cells separately and membrane potential data were combined for discussion (Shabala and Newman, 1999; Marten et al., 2010). However, there is a disadvantage to adopting this approach. The acquisition of membrane potential using a glass microelectrode requires extremely precise techniques for intracellular recordings and the experimental success rate can be low. Our proposed approach overcomes these problems by analyzing data acquired from extracellular recording and ion fluxes.

### Kinetics of Net H^+^, Ca^2+^, K^+^, and Cl^–^ Fluxes

The results of H^+^ flux measurement provided evidence that the proton pump may be involved in the electrical activities of the plant. In the present experiment, we observed that the H^+^ flux was in an efflux state for the majority of the measurement period (**Figure 5A**). This observation implied that H^+^-ATPase hydrolysis of ATP on the plasma membrane provides energy for the transport of a variety of ions and small-molecule metabolites against the electrochemical potential gradient. The H^+^ flux changed in real time in response to requirements for energy to drive transmembrane transport of various ions. In particular, H^+^ flux would inevitably increase as other ions transported across the membrane against the electrochemical concentration gradient increase, and vice versa. The sudden switch to darkness decreased the efflux of H^+^, which was associated with the gradual decrease in the efflux of K^+^ and Cl^−^ transported across the membrane against the electrochemical concentration gradient (**Figures 5A**, **7A**, and **8A**). The LIB was in a corresponding transient rising phase (see the surface potential in **Figure 9**). With extension of darkness, the H^+^ flux gradually increased, providing energy for the Ca^2+^ efflux to achieve intracellular ‘calcium homeostasis’ (Bush, 1995). At this stage, it was accompanied by K^+^ efflux and the LIB entered a falling phase. With the switch from darkness to illumination, the efflux of H^+^ instantaneously increased, which was accompanied by an increase in K^+^ efflux, and thus hyperpolarization of the plasma membranes of the population cells. Meanwhile, the surface potential showed a corresponding small decrease and then increase in response to illumination.

The efflux of H^+^ significantly decreased after 50 mM NaCl stimulation, and the duration of influx increased (**Figure 5B**). This response was probably because instantaneous salt stimulation activated the plasma membrane H^+^-ATPase pump, and H^+^-ATPase hydrolyzed ATP to generate energy to pump H^+^ out of the cytoplasm, generating a H^+^ electrochemical potential gradient across the plasma membrane, driving the plasma membrane Na^+^/H^+^ antiporter (Shabala 2000; Shi et al., 2000). Finally, H^+^ entered the cell consistent with the electrochemical potential gradient, and Na^+^ was discharged by the Salt Overly Sensitive signal-transduction pathway countering the electrochemical potential gradient (Shi et al., 2000; Shabala et al., 2005; Percey et al., 2014). The efflux of H^+^ decreased after salt stimulation, which indicated that the transmembrane ion transport against the concentration gradient was reduced, mainly because the efflux of Ca^2+^ and Cl^−^ was abolished and the K^+^ efflux decreased.

The Ca^2+^ influx is the main reason for the surface potential to enter the rising phase under illumination (**Figure 6A**). Under sudden light stimulation, the Ca^2+^ influx increased and the plasma membrane began to depolarize. As the intracellular voltage increased, the voltage-dependent anion channels were opened in about 100 s and Cl^−^ efflux was observed (**Figure 8A**). The efflux of Cl^−^ promoted further depolarization of the plasma membranes. On the basis of gray relational analysis, we observed that Cl^−^ was the most relevant ion in the depolarization phase, followed by Ca^2+^ (**Table 2**). The Ca^2+^ influx determines whether the surface potential enters a rising phase, but the efflux of Cl^−^ is the decisive factor in the rising amplitude of surface potential. The Ca^2+^ concentration in normal living cells must be maintained in a relatively stable state (“calcium homeostasis”), hence when the cytoplasmic Ca^2+^ concentration rises to a certain extent, the Ca^2+^ pump and Ca^2+^/H^+^ antiporter on the plasma membrane will continuously pump Ca^2+^ into the extracellular space or vacuole (Bush, 1995; Demidchik and Shabala, 2018). This phenomenon mainly occurred in the darkness stage.

After stimulation with 50 mM NaCl, the transmembrane transport status of Ca^2+^ changed, namely Ca^2+^ influx increased and the efflux was abolished (**Figure 6B**). This change is mainly because plants must regulate salt stress by increasing the concentration of Ca^2+^ in the cytoplasm. By comparing the extracellular Ca^2+^ concentration before and after salt stimulation, we also observed that the extracellular Ca^2+^ concentration in the leaf tissue decreased after salt stimulation, which suggested that the Ca^2+^ used for regulation of salt stress is mainly of extracellular origin. The increase in Ca^2+^ influx did not induce the surface potential to produce a larger rising phase after salt stimulation, which may be due to the equilibrium effect caused by the larger influx of Cl^−^ and efflux of K^+^ (**Figure 8B**). Mechanisms involved in the increase in intracellular Ca^2+^ concentration include regulating Na^+^ entry into the cell’s main non-selective cation channels to inhibit Na^+^ influx and restraining K^+^ efflux by inhibiting potassium outward rectifying channels (Amtmann and Sanders, 1999; Maathuis and Amtmann, 1999; White and Davenport, 2002; Shabala et al., 2006).

Potassium ions play an important role in plant electrical activity. In the study of the mechanism of action potential generation, the repolarization process is initiated when the voltage-dependent outward rectifier potassium ion channels are opened (Tsutsui et al., 1986; Koselski et al., 2017; Stolarz and Dziubinska, 2017; Kisnieriene et al., 2018). Under periodic illumination, changes in K^+^ fluxes were unique because different types of cells show different polarization states. Under sudden illumination, stomatal opening requires K^+^ uptake into guard cells, and inward-rectifying potassium channels in guard cells have been suggested to provide a pathway for K^+^ uptake into guard cells during stomatal opening. In this process, H^+^-ATPase will be activated and drastically efflux of H^+^ will occur, which causes hyperpolarization of the plasma membrane (Kwak et al., 2001; Roelfsema et al., 2001). However, for mesophyll cells and epidermal cells, transient light stimulation can depolarize the plasma membrane. At the beginning of illumination, K^+^ in mesophyll and epidermal cells will rapidly efflux through the outward potassium channel, but with the prolonged of illumination, the efflux of K^+^ will become smaller and smaller until it disappears (Elzenga et al., 1995; Shabala and Newman, 1999; Marten et al., 2010). In this paper, because there were different types of cells in the collection area of ion flux, the obtained K^+^ flux was net flux. At the beginning of illumination, the efflux of potassium ions in mesophyll and epidermal cells was greater than that in guard cells, and the net flux of K^+^ was in an efflux state. However, with the extension of the illumination time, the efflux of K^+^ of mesophyll cells decreased gradually, while the K^+^ of guard cells was stable influx, so the net flux was in an influx state. During the darkness phase, the amount of K^+^ ingested by the guard cells gradually decreases, and darkness trigger depolarization of the plasma membrane guard cells. However, the outward rectifying K^+^ channels of mesophyll cells was open and the efflux of K^+^ increased, thereafter the repolarization phase of the mesophyll is entered.

When stimulated by 50 mM NaCl, the K^+^ flux at the same position of the same leaf was significantly different from that before salt stimulation. Before salt stimulation, the net K^+^ flux was mainly in an alternating state of influx and efflux, whereas under salt stimulation the net K^+^ flux was always in an efflux state. This phenomenon has been recorded in both roots and leaves (Shabala and Newman, 2000; Cuin and Shabala, 2005). After the leaf was subjected to salt stress, although the influx of K^+^ was abolished, periodic changes in K^+^ flux were observed under periodic illumination/darkness. In addition, the magnitude of the ion flux change was greater than that in the normal condition. A reasonable explanation is that changes in membrane potential are the result of a combination of salt stimulation and periodic illumination. On one hand, under periodic illumination, when the influx of Ca^2+^ increases, it may slow the efflux of K^+^ to some extent. On the other hand, as a result of the periodic stimulation of light, the transmembrane transport state of K^+^ was constantly changing. This dynamic change occurs on the basis of a large efflux of K^+^.

As the most abundant ion in plant cells, Cl^−^ plays an important role in plant electrophysiological activities. It is generally believed that an increase in intracellular Ca^2+^ concentration will lead to the opening of the anion channels on the cell membrane, causing Cl^−^ to flow from the cytoplasm to the apoplast under the influence of the electrochemical gradient (Marten et al., 2010). In a study of the mechanism of action potential generation, a further depolarization phase was observed only when all anion channels were opened (Tsutsui et al., 1986). Under periodic illumination/darkness, changes in Cl^−^ flux also follow this rule. Under illumination, the Cl^−^ influx decreased rapidly coincident with the increase in Ca^2+^ influx (**Figures 6A** and **8A**), and then over about 100 s, Cl^−^ flux was converted from influx to efflux, and the cell membrane potential was further depolarized. With regard to the surface electrical potential, the rising phase in response to the start of illumination was also divided into two periods. The influx of Ca^2+^ in the first period is the primary force for plasma membrane polarization, whereas the main force in the second period was Cl^−^.

After stimulation by 50 mM NaCl, the influx of Cl^−^ in the broad bean leaf increased continuously under periodic illumination/darkness, and no Cl^−^ efflux phenomenon was observed (**Figure 8B**). When NaCl is used to induce stress on a plant, it is Na^+^ that has the first toxic effect on the plant. Moreover, after salt stress for a period of time, Cl^−^ will also result in a highly toxic effect on plants. This response is mainly because plants show better ability to manage Na^+^ concentrations and a weak capability to manage Cl^−^ contents (Munns and Tester, 2008).

Gray relational analysis revealed that in the surface potential rising phase under illumination, Cl^−^ was the most relevant ion followed by Ca^2+^. Indeed, the ion fluxes data show that when illumination began, Ca^2+^ influx continued to increase. This finding implied that when the cells’ membrane potential attained a certain value, the Ca^2+^-dependent anion channels may open and Cl^−^ flux would be converted from influx to efflux. The Cl^−^ with the highest GRD mainly affected the change in surface potential. After a period of illumination, the surface potential enters a falling phase; although all ion fluxes were involved in the change in surface potential, Ca^2+^ is the dominant (primary) factor impacting on surface potential. With the switch to darkness, a transient rising phase was observed. At this stage, GRD values were extremely similar, but effect of Ca^2+^ influx was stronger than that of the other ions. With extension of darkness, the surface potential entered a prolonged falling phase. The gray relational analysis showed that H^+^ efflux had the greatest influence on the prolonged falling phase, followed by K^+^. In a future study, we will calculate the GRD with salt stimulation to quantitatively investigate the dynamic relationship between ion fluxes and surface potential.

At present, the common models of plant electrical activity include action potential models (Mummert and Gradmann, 1991; Sukhov and Vodeneev, 2009; Sukhov, et al., 2011), variation transmission potential models (Sukhov et al., 2013; Vodeneev et al., 2015), a higher-plant guard cell dynamics model (Yan et al., 2009), and the Goldman–Hodgkin–Katz flux equation describing the transmembrane transport of ions (Gradmann and Hoffstadt, 1998; Gradmann, 2001a; Gradmann, 2001b). All of these models have played an important role in research on plant electrical signals. On the basis of these models in combination with data on ion fluxes under periodic illumination, a dynamic model for interpretation of electrophysiological events in plant cells will be developed in the future.

There are currently numerous useful mathematical models of electrical activity in plants (Sherstneva et al., 2016; Sukhova et al., 2017). However, challenges remain that require future investigation, e.g., the simulation of periodic illumination/darkness stimulation-induced electrical responses of higher plants under salt stress. Based on the current models (Gradmann, 2001a; Gradmann, 2001b; Shabala et al., 2005; Sukhov and Vodeneev 2009; Yan et al., 2009), the present ion fluxes and electrical potential data provide an improved understanding of the underlying mechanisms and to predict the responses to combinations of the stimuli.

## Conclusion

In this study, high reproducible LIB was obtained by a simple method, and its ion mechanism was studied by SIET technology. At the same time, the influence of various ions on LIB was analyzed by gray correlation analysis. The results showed that under periodic illumination/darkness conditions, different ions participated in surface potential changes at different stages. When the plants were subjected to different degrees of salt stress, the waveform and amplitude of LIB would change significantly. Whether the plants were subjected to salt stress according to the changes in waveform and amplitude of LIB may be determined in future research. Interpreting the mechanism of electrical activity of plants by model development is extremely challenging. We recorded periodic ion fluxes for H^+^, K^+^, Ca^2+^, and Cl^−^ and periodic surface potentials, which allowed exploration of the inter-relationships during periodic illumination and darkness stages. The relationships between ions fluxes and surface potentials provide evidence supporting the quantitative model to describe electrical activities induced by salt stimulation.

## Author Contributions

JL, HD, ZhW, XW, PH, and LH designed the research. JL, ZiW, QZ, and LF performed the research. JL, YY, LH, LF, CS, ZC, and SY analyzed the data. JL, XG, QX, and JY wrote the paper.

## Funding

This work was supported by the National Natural Science Foundation of China (grant no. 61571443).

## Conflict of Interest

The authors declare that the research was conducted in the absence of any commercial or financial relationships that could be construed as a potential conflict of interest.
