# Supplementary material for: Illumination/Darkness-Induced Changes in Leaf Surface Potential Linked With Kinetics of Ion Fluxes
Source: Front Plant Sci. 2019 Nov 7;10:1407. doi: 10.3389/fpls.2019.01407 (PMC6854870; doi:10.3389/fpls.2019.01407)
Supplement: Supplementary file 1 [file DataSheet_1.pdf]

frontiers

Linking Illumination/Darkness-induced Electrical Signal with Dynamics of Ion Fluxes

Jinhai Li[1,3], Yang Yue[1,2], Ziyang Wang[1,2], Qiao Zhou[1,2], Lifeng Fan[1,3], Zhiqiang Chai[1,3], Chao Song[1,2], Hongtu Dong[4], Shixian Yan[1,2], Xinyu Gao[1,3], Qiang Xu[1,3], Jiepeng Yao[1,2], Zhongyi Wang[1,23], Xiaodong Wang[4], Peichen Hou[4], Lan Huang[1,2*]

[1] College of Information and Electrical Engineering, China Agricultural University, Beijing 100083, China

[2] Key Laboratory of Agricultural information acquisition technology (Beijing), Ministry of Agriculture, Beijing 100083, China

[3] Key Laboratory of Modern Precision Agriculture System Integration Research, Ministry of Education, Beijing 100083, China

[4] Beijing Research Center of Intelligent Equipment for Agriculture, Beijing 100097, China

*** Correspondence:** Lan Huang (hlan@cau.edu.cn)

1 Supplementary Figures and Tables

1.1 Supplementary Figures

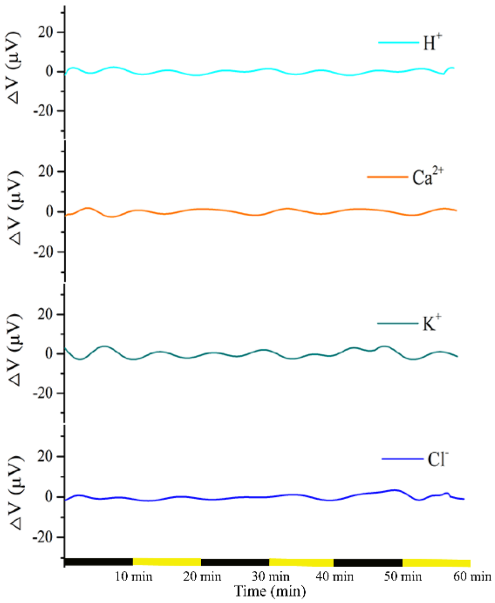

Supplementary Figure 1. The voltage difference values between the two positions of electrode under illumination/darkness without leaf sample in the base solution. For base solution, when the ion-selective electrode vibrated at a frequency of 0.5 Hz within a distance of 30 μm along the *x*-axis, there no gradient difference occurred between the two positions of the electrode.

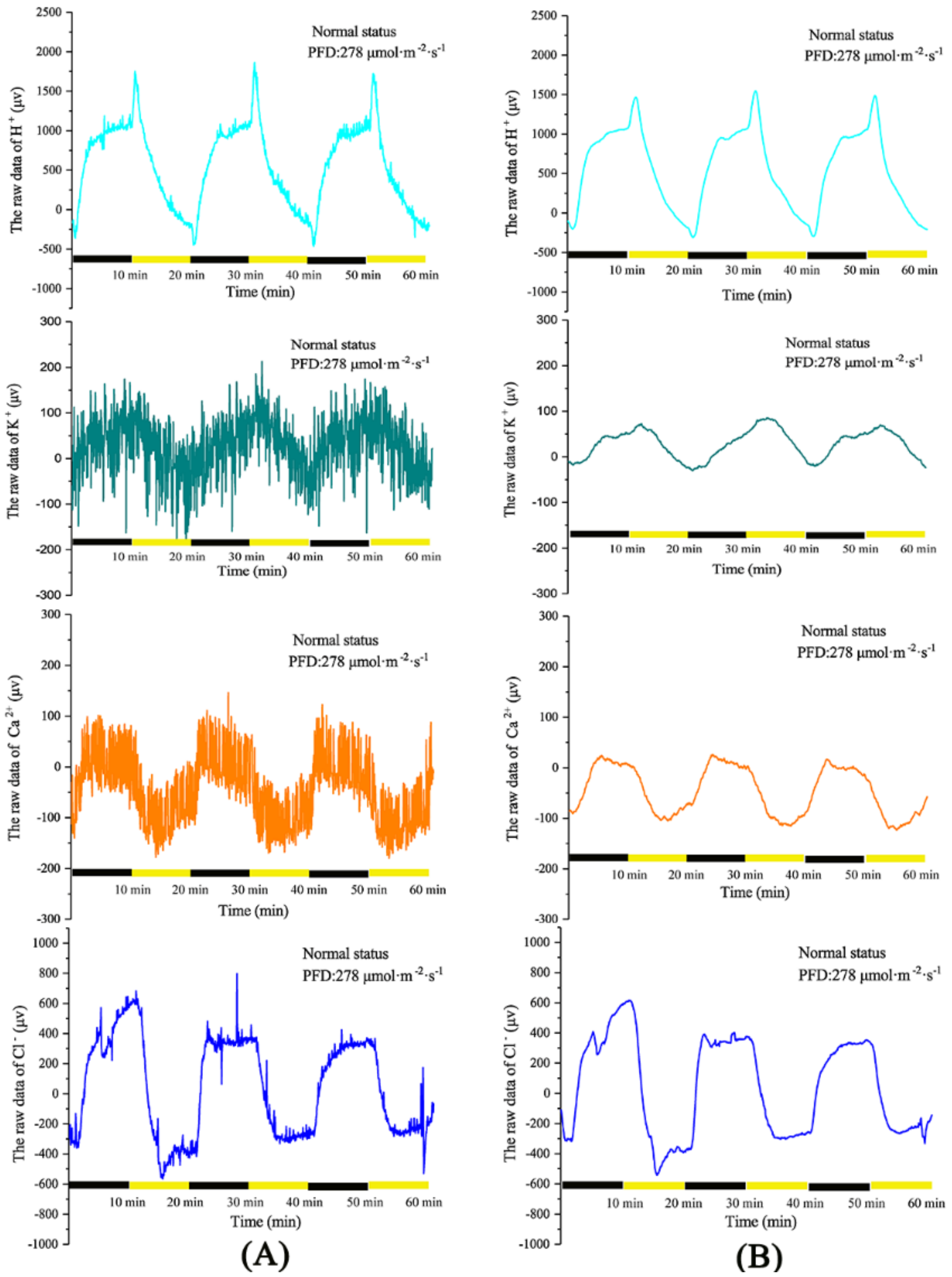

Supplementary Figure 2. Comparison of raw data before and after denoising by wavelet transform. (A) Raw data before denoising by wavelet transform. (B) Raw data after denoising by wavelet transform. Wavelet transform denoising can effectively protect useful signal spikes and abrupt signals. Therefore, wavelet transform is suitable for the noise removal of transient signals, as well as the suppression of high-frequency noise, so as to effectively distinguish high-frequency information from high-frequency noise.

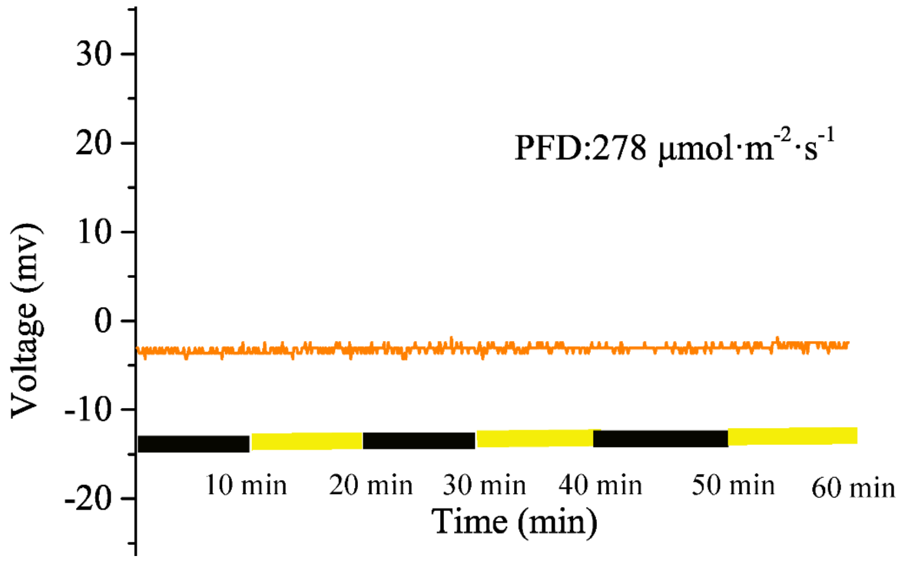

Supplementary Figure 3. The potential change without leaf under illumination/darkness. Recorded without leaf is equivalent to shorting the instrument probe to obtain a baseline voltage. Results showed that baseline is steady whether it is illumination or darkness, no potential drift will be obtained.

1.2 Supplementary Tables

Supplementary Table 1. Response time of each ion selective microelectrode in this work

		Response time/ms								
		1	2	3	4	5	6	7	8	Max
H^+	pH 5.5	340	360	290	260	380	250	290	350	380
	pH 6.86	380	390	270	380	300	350	350	340	390
Ca^{2+}	1 mM	320	360	340	430	320	400	410	320	430
	10 mM	330	330	330	340	400	320	400	260	400
K^+	1 mM	200	240	190	200	200	230	210	250	250
	10 mM	250	230	270	300	240	300	280	220	300
Cl^-	2 mM	320	200	250	210	190	200	230	180	320
	20 mM	350	270	210	220	190	300	330	260	350

Supplementary Table 2. Ionic mechanisms of light-induced bioelectrogenesis

Illumination	Stage (Figure 9)	Data description	Explanation and discussion
Compared with the baseline, the surface potential decreased briefly and thereafter returned to the initial value.	0–1	Ca^{2+} and Cl^-: influx<br><br>K^+ and H^+: efflux	Changes in surface potentials were mainly caused by the dramatic increase in the efflux of H^+.<br><br>It is generally considered that hyperpolarization occurs in guard cells, which is caused by the influx of K^+ (Marten et al., 2010).<br><br>However, on the basis of the present results, the net K^+ flux was in an efflux state. This phenomenon may be the result of K^+ efflux from the mesophyll cells that was greater than the influx from the guard cells. The instantaneous increase in H^+ efflux induced changes in surface potential.
Compared with the baseline, the surface potential was in a rising phase.	1–2	Ca^{2+}: influx increased<br><br>Cl^-: influx but decreased at a faster rate<br><br>K^+ and H^+: efflux increased	Although the efflux of H^+ increased, depolarization in this stage gradually activated the outward potassium channels and thus increased the efflux of K^+. Ca^{2+} influx increased and K^+ efflux increased. The large influx of Ca^{2+}, which increased intracellular voltage and depolarized the plasma membranes, was the primary reason for the surface potential being in a rising phase.
	2–3	H^+: efflux decreased<br><br>Ca^{2+}: influx increased<br><br>Cl^-: changed to efflux	Although Ca^{2+} influx continued to increase in this stage, the large Cl^- efflux was the main reason for further depolarization of plasma membranes. Before the Cl^- efflux, the outward potassium channels were further opened and repolarization of plasma membranes began.

		K^+: efflux increased significantly	
Compared with the baseline, the surface potential was in a falling phase.	3–4	Ca^{2+}: influx decreased<br>K^+: efflux decreased<br>Cl^-: steady efflux<br>H^+: efflux decreased	K^+ and H^+ were in an efflux state, which participated in the repolarization of the plasma membranes.

**Darkness**	**Stage (Figure 9)**	**Data Description**	**Explanation and discussion**
Compared with the baseline, the surface potential was in a temporary rising phase.	4–5	Cl^-: efflux<br>Ca^{2+}: influx<br>K^+: stable efflux<br>H^+: efflux decreased	This stage was consistent with many previous studies (Felle et al., 2000; Zivanovic et al., 2005; Percey et al., 2016). The efflux of K^+ was mainly exhibited by the guard cells and mesophyll cells. Ca^{2+} influx maintained the activity of anion channels that caused the efflux of Cl^-. The efflux of K^+ caused hyperpolarization in the guard cells. K^+ efflux occurred in mesophyll cells.
Compared with the baseline, the surface potential was in a prolonged falling phase.	5–6	Cl^-: efflux<br>Ca^{2+}: influx<br>K^+ and H^+: efflux decreased	The efflux of H^+ decreased to the minimum value, which indicated the transmembrane transport that inverted the concentration gradient of Cl^- and K^+ was also reduced in this stage. In the meantime, Ca^{2+} influx decreased. The surface potential entered a prolonged falling phase.
	6–7	Cl^-: influx increased Ca^{2+}: efflux<br>K^+: efflux decreased H^+: efflux increased	Consistent with previous studies (Felle et al., 2000; Zivanovic et al., 2005). Under darkness, K^+ was in an efflux state in mesophyll cells, which maintained the activity of extracellular K^+ and made it difficult for guard cells to lose potassium.

Darkness	Stage (Figure 9)	Data Description	Explanation and discussion
	7–8	Cl^-: stable influx, Ca^{2+}: efflux<br>K^+: influx<br>H^+: efflux increased	The influx of Cl^- and efflux of Ca^{2+} were both involved in the repolarization of the plasma membrane, but H^+ and K^+ were the primary forces of the repolarization of population cells.
The surface potential continued to decrease after attaining the initial value and then was gradually restored to the initial value.	8–9	K^+: turned into efflux<br>Cl^-: stable influx<br>H^+: efflux increased<br>Ca^{2+}: efflux	K^+ and H^+ were in an efflux state. The proton pumps may participate in hyperpolarization of plasma membranes and maintained the negative potential of the cell membrane.

2 References

Felle, H. H., et al. (2000). Dynamics of ionic activities in the apoplast of the sub-stomatal cavity of intact Vicia faba leaves during stomatal closure evoked by ABA and darkness. Plant Journal 24(3): 297-304.

doi: 10.1046/j.1365-313x.2000.00878.x

Marten, I., et al. (2010). Light-induced modification of plant plasma membrane ion transport. Plant Biology 12: 64-79.

doi: 10.1111/j.1438-8677.2010.00384.x

Percey, W. J., et al. (2014). Ion transport in broad bean leaf mesophyll under saline conditions. Planta 240(4): 729-743.

doi: 10.1007/s00425-014-2117-

Zivanovic, B. D., et al. (2005). Light-induced transient ion flux responses from maize leaves and their association with leaf growth and photosynthesis. Plant Cell and Environment 28(3): 340-352.

doi: 10.1111/j.1365-3040.2005.01270.x
